# Traumatic Ulcerations of the Tongue

**Published:** 1902-10-11

**Authors:** 


### TRAUMATIC ULCERATIONS OF THE TONGUE.

In a clinical lecture delivered at St. George's Hospital Mr. Marmaduke Sheild,[1] dealing with the wide subject of ulcers of the tongue, made some remarks concerning traumatic ulcers which are worthy of careful consideration. He said that the traumatic ulcer of the tongue was of surpassing importance in that it was so often overlooked in practice. Nothing, he said, was more frequent than for an ulcer of the tongue to form from injury, that injury being almost invariably produced by a "jagged" tooth, part of which has been broken off, a rough edge being left against which the tongue is perpetually abraded. A badly fitting tooth plate is equally serious as a cause of these traumatic ulcerations. An injury so caused may develop into an ulcer so like cancer that it is very difficult to diagnose between the two conditions, and in elderly people such ulcers, if neglected, almost invariably become cancers. Rarer forms of traumatic ulcers of the tongue are those produced by scalds from hot fluids, or wounds from pieces of fish bone and the like. The burning and friction caused by the stem of a clay pipe may act in the same injurious manner. A characteristic of this class of ulcers is their position at the side of the tongue and the detection of the exciting cause. The extraction of a rough tooth may cause the rapid healing of a very dangerous-looking ulcer. "Never," he says, "irritate such sores by rubbing them with caustics. This treatment only precipitates the danger of cancer. It is the worst thing that you can do."

[1] The Clinical Journal, Sept. 24.

